# Congestion in Heart Failure: From the Secret of a Mummy to Today’s Novel Diagnostic and Therapeutic Approaches: A Comprehensive Review

**DOI:** 10.3390/jcm13010012

**Published:** 2023-12-19

**Authors:** Ioannis Alevroudis, Serafeim-Chrysovalantis Kotoulas, Stergios Tzikas, Vassilios Vassilikos

**Affiliations:** 1 Third Department of Cardiology, Ippokratio General Hospital, Aristotle University of Thessaloniki, 54642 Thessaloniki, Greecevvas63@otenet.gr (V.V.); 2 Intensive Care Medicine Clinic, Ippokratio General Hospital, Aristotle University of Thessaloniki, 54642 Thessaloniki, Greece; akiskotoulas@hotmail.com

**Keywords:** acute decompensated heart failure, fluid overload, loop diuretics, diuretic resistance, mineralocorticoid, SGLT2 inhibitors, ultrafiltration

## Abstract

This review paper presents a review of the evolution of this disease throughout the centuries, describes and summarizes the pathophysiologic mechanisms, briefly discusses the mechanism of action of diuretics, presents their role in decongesting heart failure in patients, and reveals the data behind ultrafiltration in the management of acutely or chronically decompensated heart failure (ADHF), focusing on all the available data and advancements in this field. Acutely decompensated heart failure (ADHF) presents a critical clinical condition characterized by worsening symptoms and signs of heart failure, necessitating prompt intervention to alleviate congestion and improve cardiac function. Diuretics have traditionally been the mainstay for managing fluid overload in ADHF. Mounting evidence suggests that due to numerous causes, such as coexisting renal failure or chronic use of loop diuretics, an increasing rate of diuretic resistance is noticed and needs to be addressed. There has been a series of trials that combined diuretics of different categories without the expected results. Emerging evidence suggests that ultrafiltration may offer an alternative or adjunctive approach.

## 1. Introduction and Background

Annual heart failure hospitalizations exceed 1 million in both the United States and Europe, and more than 90% are due to symptoms and signs of fluid overload. Additionally, up to one in four patients (24%) are readmitted within 30 days, and one in two patients (50%) are readmitted within 6 months [1]. Acute decompensated heart failure (ADHF) remains the leading cause of hospitalization in patients > 65 years old and has the highest rate of 30-day rehospitalization among all medical conditions [2]. Recurrent fluid overload in heart failure has been associated with worse outcomes independently of age and renal function [3]. Deranged hemodynamics, neurohormonal activation, excessive tubular sodium reabsorption, inflammation, oxidative stress, and nephrotoxic medications are important drivers of harmful cardiorenal interactions in patients with heart failure [4]. Central venous pressure elevation is rapidly transmitted to the renal veins, causing increased interstitial and tubular hydrostatic pressure, which decreases net glomerular filtration [5]. Venous congestion itself can produce endothelial activation, the up-regulation of inflammatory cytokines, hepatic dysfunction, and intestinal villi ischemia [6]. Thus, the foremost goal in managing acutely decompensated heart failure is to effectively resolve fluid overload [7].

## 2. Heart Failure: Pathophysiology and Classification

Heart failure represents a clinical syndrome that consists mainly of symptoms like shortness of breath, orthopnea, ankle swelling, and fatigue and can be accompanied by signs of congestion like increased central venous pressure, pulmonary crackles, or lower-limb edema [8]. It can be the result of either a structural or functional abnormality leading to decreased cardiac output, increased intraventricular pressure, and decreased tolerance to exercise. Coronary artery disease and diabetes mellitus have become the predominant predisposing factors for heart failure. Other structural causes of congestive heart failure (CHF) include hypertension, valvular heart disease, uncontrolled arrhythmia, myocarditis, and congenital heart disease. Finally, diastolic heart failure with impaired ventricular filling can also be caused by restrictive cardiomyopathy and constrictive pericarditis [9]. A rapid increase in blood pressure (afterload), particularly in patients with diastolic dysfunction, may precipitate severe pulmonary congestion.

In recent years, interest has fallen on the right ventricle (RV) and how it can participate in the phenotype of HF. It is well known that a failing left ventricle (LV) can lead to an increase in the pulmonary capillary wedge pressure (PCWP) and transpulmonary pressure and eventually increase the afterload of the RV. This increase in pressure will lead to RV distention as a response mechanism in order to maintain adequate cardiac output. This distention will eventually affect contractility to aggravate tricuspid regurgitation, increase ventricular interdependence, impair LV filling and cardiac output (CO) reduction, and multi-organ dysfunction [10]. In fact, a recent retrospective study that included six hundred and seventy-seven severely ill patients with acute COVID-19 patients admitted to an ICU showed that one-third of those presented right ventricle systolic dysfunction. This presentation was attributed to positive mechanical ventilation with high positive end-expiratory pressure (PEEP) due to severe ARDS, hypercapnia, and pulmonary embolism [11]. One of the major factors causing PE is the significant increase in the capillary hydrostatic pressure of the pulmonary circulation, following the Frank–Starling law. Normal pulmonary circulation is a system of high flow, low resistance, and low pressure under normal conditions. Factors that cause abnormal pulmonary circulation will eventually cause a mismatch between the right heart and the flow in the circulatory bed leading to PE. Considering the role of the pressure generated by the right ventricle in maintaining the capillary hydrostatic pressure, it is understood that its failure will lead to congestion [12].

In spite of the great variety of clinical profiles and the heterogeneity of the underlying cause of HF, the majority of patients with AHF will present signs and symptoms of pulmonary congestion with or without systemic congestion. This presentation may not be connected to decreased cardiac output (CO). Congestion will lead to dyspnea, which is the major symptom among patients with AHF [13]. However, the initiation of diuretic treatment might not always lead to dyspnea relief [14]. Moreover, increased PCWP is not always associated with dyspnea severity, in such a manner that high PCWP may cause mild dyspnea, while lower PCWP can cause severe dyspnea [15].

An imbalance between the forces that drive fluid into the alveoli and the removal mechanism, leads to pulmonary edema. Two fundamental processes may lead to alveolar-capillary barrier dysfunction in AHF: (a) mechanical injury of the barrier due to increased hydrostatic pulmonary capillary pressures and (b) inflammatory and oxidative lung injury.

Pulmonary endothelium can induce several intracellular signaling pathways, leading to increased inflammatory cytokine production, macrophage activation, acute inflammation, and barrier dysfunction [16]. This oxidative and inflammatory lung injury further damages the alveolar-capillary barrier and increases its permeability leading to a decrease in pulmonary fluid accumulation by the capillary hydrostatic pressure. This could partially explain the recurrence that the AHF patients present.

Another cause of acute decompensation of HF is hypoalbuminemia, which results in low serum colloid osmotic pressure (COP), facilitating the onset of pulmonary edema in patients with diastolic heart failure (DHF). Reduced COP allows more fluid to leak out of the blood vessels, while elevated PAWP indicates increased pressure in the pulmonary capillaries, promoting the movement of fluid into the lung tissue. Hypoalbuminemia, as a sign of cachexia, can be present in HF patients during the evolution of the disease and is exacerbated when other organs become involved, like the liver, due to congestion.

It has been shown that a major role in the decompensation of Heart failure is attributed to inadequate drug treatment, failure to comply with the dietary sodium restriction, and decreased physical activity [17].

In the primary stages of congestive heart failure, the heart muscle uses several compensatory mechanisms in order to maintain cardiac output in an attempt to keep up with the systemic demands. These mechanisms include changes in myocyte regeneration, myocardial hypertrophy and hypercontractility, and the Frank–Starling mechanism, which increases cardiac output. The increasing wall stress will force the myocardium to compensate via eccentric remodeling, leading to fibrosis and eventually affecting the loading conditions and wall stress [17].

The most commonly used heart failure classification is based on the left ventricle ejection function. The rationale behind this old classification is based on the fact that treatment has a bigger benefit to the lowest ejection fraction [18] (Table 1).

Heart failure can present either as a chronically decompensated status (CHF), where the diagnosis is set and the symptoms build up throughout the years of the disease evolution, or as an acute decompensation, which could lead to a decrease in the cardiac output either at a rapidly or slowly evolving pace. These two types necessitate the use of a decongestion treatment either in a conservative form with the use of diuretics alone or with the aid of ultrafiltration.

Finally, NYHA classification categorizes heart failure patients according to their functional status starting from class I, where the patient is almost completely functional, up to class IV, where the patient has reached the last stage of the disease and, unless transplanted or mechanically supported, will have very poor prognosis (Table 2).

### 2.1. Fluid Overload Significance

Fluid overload (FO) is a crucial and often central aspect of heart failure (HF) for several reasons. Understanding its importance in HF is fundamental to managing the condition effectively. Here are some key points highlighting its significance:

1. Symptom Severity: Fluid retention is a primary contributor to the hallmark symptoms of heart failure, such as dyspnea (shortness of breath), edema (swelling), and weight gain. As fluid accumulates in the lungs and peripheral tissues, the heart begins to fail, followed by the kidneys. The kidneys respond by increasing the production of renin, leading to more aldosterone production, which is consequently followed by sodium and water retention [19]. In some patients, pulmonary congestion evolves rapidly because of a sudden increase in LV filling pressures. A precipitating factor is often recognized, like acute myocardial ischemia, or uncontrolled hypertension. In this instance, the edema is mainly present in the pulmonary airspaces (pulmonary edema), while the total amount of fluid in the cardiovascular system remains unchanged [20].
2. Hemodynamic Disturbances: The accumulation of excess fluid in the body increases the blood volume and venous pressure, resulting in intravascular and interstitial fluid volume expansion and redistribution. This, in turn, leads to elevated preload and afterload, negatively affecting cardiac function. Increased preload can worsen the workload of the heart muscle, further compromising its pumping efficiency. It has been described using the concept of fluid redistribution, which suggests that multiple factors like myocardial ischemia, episodes of high blood pressure, failure to comply with the pharmaceutical regimen, worsening renal function, and increased neurohormonal-sympathetic activation could increase the venous tone and decrease the venous capacitance, which, in the setting of existing intravascular volume overload, could only redistribute fluid from a peripheral venous reservoir like the splanchnic venous bed to the central cardiopulmonary circulation [21]. This results in the production of transudate fluid in the pulmonary alveolar space and the development of worsening dyspnea and symptomatic clinical congestion. This acute translocation of as much as 1 L of fluid, which will not alter the net body weight, will cause pulmonary congestion and contribute to the overall discomfort experienced by HF patients [22].
3. Reduced Cardiac Output: Initially, compensatory mechanisms attempt to maintain cardiac output to meet systemic demands. These include myocardial hypertrophy, hypercontractility, apoptosis, and the regeneration of myocardial cells. The increased wall stress will lead to eccentric remodeling that further aggravates the loading conditions of the heart [23]. Due to decreased cardiac output, the neuroendocrine system takes over releasing epinephrine, norepinephrine, endothelin-1 (ET-1), and vasopressin. The resulting vasoconstriction will lead to increased afterload, which, together with the increased levels of cyclic adenosine monophosphate (cAMP) and cytosolic calcium in myocytes, will further inhibit the myocardial muscle from relaxing. The oxygen demand in the myocardium increases, necessitating a further increase in cardiac output, leading to myocardial cell and apoptosis. The decreasing cardiac output will stimulate the renin–angiotensin–aldosterone system (RAAS), leading to increased salt and water retention, along with increased vasoconstriction. Moreover, RAAS releases Angiotensin II, which is shown to increase myocardial cellular hypertrophy and interstitial fibrosis. This maladaptive function of angiotensin II increases myocardial remodeling [24]. This reduction in cardiac output can lead to inadequate oxygen delivery to the body’s tissues, causing fatigue and exercise intolerance.
4. Kidney Function: Fluid retention can also impact kidney function. Inflammation and ischemia-reperfusion injury will lead to endothelial injury and fluid overload, damaging the endothelial glycocalyx (EGL) and causing capillary leakage. This leakage will lead to interstitial edema and a reduction in the circulating intravascular volume since the volume of the interstitial compartment will be lost. This interstitial edema is the cause behind acute kidney injury (AKI), as well as progressive organ failure, due to the blockage of lymphatic drainage and the poor interaction between cells [25]. Finally, fluid overload causes atria distention and the stretching of vessel walls, causing the release of ANP and further damage to the EGL, aggravating the AKI [26]. This is a key contributor to the development of diuretic resistance, which is a common challenge in managing HF. Renal congestion increases renal tubular pressure, reducing the glomerular filtration rate (GFR) and diuresis.
5. Electrolyte Imbalances: Fluid overload and diuretic therapy can lead to electrolyte imbalances, most commonly hyponatremia, hypokalemia, and hypomagnesemia [27]. The acid–base disturbances generally observed are metabolic alkalosis, either pure or combined with respiratory alkalosis [28]. Hyponatremia, which is the most common electrolyte abnormality observed in hospitalized subjects, is defined as a serum sodium concentration lower than 136 mmol/L [29]. Mild-to-moderate hyponatremia is generally present in 10% of HF patients [30]. In the OPTIME-CHF trial, 27% of patients had serum sodium concentrations between 132 and 135 mEq/L [31], while in the ESCAPE trial, persistent hyponatremia, defined as serum sodium below 134 mEq/L, was present in 18% of the hospitalized patients. Hypokalemia is commonly observed in CHF patients, and it is a strong independent predictor of mortality [32]. Hypokalemia has not been well defined in HF, and even in the literature, its range varies from 3.5 to 4.0 mEq/L (mmol/L) [33]. Hypokalemia is generally more evident in patients with advanced CHF receiving intensive diuretic therapy and those whose renin–angiotensin system is highly activated [34]. Low levels of serum K+ may be a marker of increased neurohormonal activity and disease progression [35]. Diuretics and adrenergic stimulation may cause hypokalemia, while neurohormonal blockade using ACE inhibitors, angiotensin receptor blockers, beta-blockers, and aldosterone antagonists may cause hyperkalemia. These drug effects require frequent control of K+ in these patients [36]. The prevalence of hypomagnesemia in CHF subjects ranges from 7% in well-compensated ambulatory patients to 52% in more advanced CHF patients who are under aggressive diuretic treatment [37]. Magnesium deficiency in animal models alters the mitochondrial structure with calcium accumulation, cell death, and multifocal myocardial necrosis [38]. There is confirmation that the effective correction of magnesium disturbances is favorable in CHF patients [39], mostly due to the reduction in potentially lethal arrhythmias. Diuretics (loop-acting diuretics in particular) produce most of the renal magnesium loss, especially in the volume-expanded setting of CHF [40].
  Few cases of hypocalcemia (total serum calcium concentration < 8.6 mg/dL or ionized calcium concentration < 1.1 mmol/L) in CHF have been reported and are often associated with hypomagnesemia [28]. Loop diuretics block the reabsorption of calcium in the loop of Henle and may play a role in the pathogenesis of hypocalcemia [41]. The correction of a calcium disorder could improve CHF [42]. These imbalances can cause cardiac arrhythmias and muscle weakness, complicating the clinical picture in HF.
6. Mortality Risk: The severity of fluid retention is often linked to the prognosis of HF. Patients with more significant fluid overload tend to have a higher risk of mortality. Addressing fluid retention is, therefore, essential for improving patient outcomes.
7. Hospitalizations, readmissions, and Quality of Life (QoL): Effective management of fluid overload can significantly enhance a patient’s quality of life. The rehospitalization rate is a comprehensive measure of disease burden and progression. While the length of hospital stay has decreased over time in heart failure patients, readmission rates have essentially remained unchanged [43]. Congestion is the most frequent cause of readmission. Other factors associated with increased risk of readmission include higher age, comorbidities, premature discharge, and noncompliance. Hospitalization is easy to identify and easy to quantify. Early readmission is associated with worse long-term outcomes and significant increases in heart-failure-related health costs. With each readmission, QoL declines [44].

In summary, fluid overload is central to the pathophysiology and clinical presentation of heart failure. It impacts symptoms, cardiac function, kidney function, and overall prognosis. Therefore, the effective management of fluid is a cornerstone of heart failure treatment, emphasizing the need for an integrated approach that includes diuretics and other therapeutic interventions to address this critical aspect of the condition.

### 2.2. Congestion and Extracellular Fluid Overload (FO) Assessment

It is of paramount importance to recognize and treat fluid overload (FO) since early treatment can prevent or ameliorate the adverse events caused by the extra volume buildup. There are markers to monitor in order to reveal and quantify the extra volume overload, including inflammatory markers (C-reactive protein, myeloperoxidase), markers suggestive of fibrosis and extracellular remodeling (procollagen, ST2, galectin-3), markers for mechanical strain/stretch (natriuretic peptides, CD146, carbohydrate antigen 125 [CA125]), and markers of hemodynamic homeostasis (copeptin, adrenomedullin), tissue perfusion (lactate), and heart muscle injury (troponins) [45].

There is a clear association between the decrease in the red blood cell concentration and plasma volume expansion. Increasing values of hematocrit have been suggested as a surrogate for the plasma refill rate and decongestion rate [46]. Fujita et al. found that hemodilution during the first 3 days of hospitalization in patients with acute heart failure was associated with both increasing rates of pulmonary edema in comparison to those with hemoconcentration (85 vs. 63%, *p* < 0.01) and the HF-related readmission rate (34 vs. 9%, *p* < 0.01) [47]. In the PROTECT (Placebo-Controlled Randomized Study of the Selective Adenosine A1 Receptor Antagonist Rolofylline for Patients Hospitalized with Acute Decompensated Heart Failure and Volume Overload to Assess Treatment Effect on Congestion and Renal Function) study, the rapid increase in the hemoglobin concentration during the first 7 days of hospitalization in patients presenting with acute decompensated heart failure was associated with a favorable outcome, despite the incidences of acute kidney injury (AKI) that were observed [48]. Low hemoglobin levels, however, should be interpreted with caution in order not to mistake dilution-related pseudo-anemia for true anemia, especially if under erythropoietin treatment [49].

Moreover, echocardiography is a useful tool in assessing the overall function of the heart. In the case of chronically decompensated heart failure, FO leads to pressure overload, which flattens the interventricular septum both in diastole and systole [50]. If the pressure overload occurs acutely, the septum is pushed during diastole, causing the characteristic D-shaped left ventricle. The tricuspid regurgitation caused by right ventricular enlargement will increase both the right atrial pressure and the central venous pressure (CVP) [51]. A post-hoc analysis of the ECHO-COVID study in severely ill COVID-19 patients hospitalized in intensive care units (ICU) who underwent two echocardiographic evaluations found that the patients presenting signs of acute cor pulmonale (RV dilatation with paradoxical septal motion) in their last critical care echocardiography had worse outcome. Other echocardiographic phenotypes included RV failure with dilatation and congestion, as well as RV dysfunction with a tricuspid annular plane systolic excursion (TAPSE) equal to or smaller than 16 mm [52]. Inferior vena cava (IVC) ultrasound will reveal the diameter of the vessel and whether there is an IVC collapse during respiration. Volume depletion is considered with an IVC diameter of <1.5 cm while an IVC diameter of >2.5 cm suggests volume overload. An IVC diameter of ≤2.1 cm and collapsibility of >50% with a sniff indicates normal RAP of 3 mm Hg (0–5 mm Hg), an IVC diameter of >2.1 cm with <50% inspiratory collapse indicates high RAP of 15 mm Hg (10–20 mm Hg), and scenarios in between correspond to an intermediate value of 8 mm Hg (5–10 mm Hg) [53]. The IVC respiratory variation is a feasible, easily reproducible examination in most patients. However, the standard subcostal (SC) view is not always possible due to obesity, enlarged bowels, and recent abdominal surgery. These are the cases where the trans-hepatic view could be used as an alternative approach. However, there is limited evidence regarding the interchangeability of TH and SC views. This issue can be tackled by the use of AI with automated border tracking. In a recent study, 66 patients were included to visualize IVC. In five of them, visualization was not possible. In these cases, AI showed good accuracy for both the SC and TH approaches. The correlation between SC and TH assessments was poor for M-mode (ICC = 0.08 [−0.18; 0.34]) and moderate for AI (ICC = 0.69 [0.52; 0.81]) [54].

The increase in the right atrial pressure is also transmitted, through liver sinusoids, into the portal vein. While normal portal flow is continuous or only mildly pulsatile, in the case of increased atrial pressure, there is increased pulsatility in portal venous flow [55]. This increase can be found in patients with increased systolic pulmonary pressure [56], right ventricular dysfunction [57], and increased intravascular volume [58]. Finally, a rise in NT-proBNP seems to be correlated with a rise in portal vein pulsatility.

While the chest X-ray, historically the first diagnostic tool in doctors’ diagnostic algorithm, can show signs of lung congestion and pleural fluid, 20% of patients with congestion exhibit a normal chest X-ray [59]. The lung ultrasound (LUS) has evolved over recent years as a trustworthy tool for ruling out interstitial edema and pleural effusions. LUS uses a high-frequency probe (7–12 MHz) to achieve a better resolution. The examination is typically performed with the patient in a sitting or semi-recumbent position. The thorax is divided into different regions, and each region is systematically scanned. A linear or phased-array probe is moved along intercostal spaces to visualize the pleural line and underlying lung parenchyma.

In a normally aerated parenchyma, the beam is scattered, and no structure can be visualized. The only exception to this is the pleural line that produces the characteristic reverberation artifact of a hyperechoic horizontal line (i.e., A-lines). A-lines are formed due to multiple reflections of the bean from the interface of the air filling the alveoli under the pleura and the soft tissues of the chest wall [60]. It detects B-lines originating from extravasated fluid into the interstitium and alveoli. B-lines represent the reflection of the US beam by thickened subpleural interlobular septa [61]. The presence of more than three B-lines in more than two intercostal spaces bilaterally is thought to be sufficient in order to detect interstitial and alveolar edema in acute heart failure. The sensitivity reaches almost 90%, but it lacks specificity since the described interstitial edema can have a non-cardiac origin. However, the rapid response to the diuretic treatment and the reduction in the b-lines suggest that the decision of decongestion was correct. In patients admitted with acute dyspnea, pulmonary congestion proven by b-lines is significantly correlated to NT-proBNP values, making it an accurate diagnostic tool for the origin of the dyspnea [62].

Many trials tried to guide FO severity, in-hospital treatment, and the discharge strategy according to LUS findings. The lung ultrasound-guided treatment in ambulatory patients with heart failure (LUS-HF9) single blinded clinical trial included 123 patients who were admitted for HF and randomized to either a standard follow-up (*n* = 62, control group) or an LUS-guided follow-up (*n* = 61, LUS group). The primary endpoint was a composite of urgent visits, hospitalization for worsening HF, and death from any cause. Hospitalization for worsening HF was defined as a stay in hospital for >24 h mainly as a result of signs and/or symptoms of worsening HF. Even though patients in the LUS group received more loop diuretics [51 (91%) vs. 42 (75%); *p* = 0.02] and showed an improvement in the distance achieved in the 6-min walking test [60 m (interquartile range: 29–125 m) vs. 37 m (interquartile range: 5–70 m); *p* = 0.023], they showed no benefit in mortality or rehospitalizations [63]. The Efficacy of Lung Ultrasound Guided Therapy to Prevent Rehospitalizations in Heart Failure (CLUSTER-HF) was another single-center, single-blind, randomized controlled trial that showed similar LUS protocols and results to the LUS-HF trial [64]. Finally, the LUST trial randomized 367 patients at high cardiovascular risk (history of coronary artery disease and/or New York Heart Association (NYHA) class III-IV heart failure) to an LUS-guided intervention vs. standard clinical care [65]. The majority of HF patients were diagnosed with HFpEF. The primary endpoint of this study was a composite of death, myocardial infarction, or newly diagnosed decompensated heart failure. Even though pulmonary congestion subsided, the LUS-guided intervention did not result in a lower probability of the primary endpoint.

Equally sensitive but less specific is the mitral inflow E-wave velocity, with a value of >50 (cm/s) suggestive of pulmonary capillary wedge pressure (PCWP) > 18 mmHg. The deceleration time of the mitral valve has both high specificity and sensitivity, reaching 80%, but is affected by the same weakness in the mitral inflow E-wave velocity, which is a more difficult diagnosis when there is fusion of the E and A waves [66].

### 2.3. Pleural Effusion

HF leads to pleural effusion formation due to an increase in pulmonary capillary pressure and a consequent leak into the pleural space. Of those patients with decompensated heart failure requiring diuretic treatment, 87% have pleural effusions on CT [67]. Patients with uncomplicated heart failure with pleural effusions have bilateral effusions in 73% of cases [68]. The process of accumulation of the fluid is thought to be due to processes that include decreased pleural fluid reabsorption, increased hydrostatic pressure in bronchial veins, or dilated pulmonary arteries obstructing lymphatic flow. In view of the dyspnea associated with heart failure itself, patients may benefit considerably from the drainage of effusions that persist despite optimal heart failure treatment. Therapeutic aspiration fulfills an important role in the management of any pleural effusion. At the time of obtaining a pleural fluid sample for diagnostic purposes, a substantial volume may be removed for therapeutic purposes. This should improve patient symptoms during the period in which diagnostic evaluation may occur. Moreover, it allows evaluation of the time that fluid builds up, provides evidence of the degree of symptomatic improvement that can be gained through the removal of fluid, and clarifies whether there is any evidence of trapped lung, which may direct future management.

Even though many methods have been proposed to distinguish exudate from transudate liquid, Light’s criteria have remained the standard method of calculation for the past 40 years. The original description had a sensitivity to rule out exudates of 99% and a specificity to rule out exudates of 98% [69] (Table 3).

However, there is evidence suggesting the misclassification of transudates using Light’s criteria usually in patients with heart failure or cirrhosis who take diuretics (up to one-third of patients) [70]. To tackle this, further examination of the fluid seems to be reasonable. Measurement of the NT-proBNP level in pleural fluid seems to be accurate in diagnosing the etiology of the effusion as CHF. Pleural fluid levels above 2220 pg/mL show high diagnostic sensitivity suggesting CHF [71].

In some patients, therapeutic aspiration may represent a legitimate therapeutic option in the long term. Depending on the speed at which fluid re-accumulates, therapeutic aspirations may only be required at a frequency acceptable to both clinicians and patients. Lung ultrasound-driven therapeutic thoracentesis of pleural effusion in decompensated heart failure patients appears to be safe and efficient. It induces, as it is anticipated, fast and substantial symptomatic relief followed by long-lasting improvement [72].

### 2.4. Diuretic Agents

Diuretics are medications that promote diuresis, commonly used to treat conditions such as hypertension, heart failure, and edema (fluid retention). There are several classes of diuretics, each with its mechanism of action and specific indications. The main classes are as follows: loop diuretics, thiazide diuretics, potassium-sparing diuretics, and carbonic anhydrase inhibitors (Table 4 and Table 5).

#### 2.4.1. Loop Diuretics (LD)

- Mechanism of Action: Loop diuretics act on the thick ascending limb of the loop of Henle in the nephron of the kidney. They inhibit the reabsorption of sodium and chloride ions, leading to increased diuresis.
- Indications: Loop diuretics are potent and are often used in the treatment of acute and severe conditions of fluid overload, such as acute heart failure, pulmonary edema, and edema associated with renal dysfunction.
- Common Medications: Examples of loop diuretics include furosemide, torsemide, and bumetanide.

#### 2.4.2. Thiazide Diuretics (THZ)

- Mechanism of Action: Thiazide diuretics act on the distal convoluted tubules of the nephron. They inhibit sodium and chloride reabsorption, leading to increased urine production.
- Indications: Thiazide diuretics are typically used in the management of hypertension and mild to moderate edema. They are also sometimes used in the treatment of certain kidney stone conditions, such as calcium oxalate stones.
- Common Medications: Examples of thiazide diuretics include hydrochlorothiazide, chlorthalidone, and indapamide.

#### 2.4.3. Potassium-Sparing Diuretics (MRA)

- Mechanism of Action: Potassium-sparing diuretics act on the distal tubules and collect ducts of the nephron. They promote diuresis while minimizing potassium excretion. Some potassium-sparing diuretics work by blocking the action of aldosterone, a hormone that typically promotes sodium and water retention while increasing potassium excretion.
- Indications: Potassium-sparing diuretics are often used in combination with other diuretics to help counteract the potassium loss associated with loop and thiazide diuretics. They are also used in conditions where retaining potassium is important, such as hypokalemia.
- Common Medications: Examples of potassium-sparing diuretics include spironolactone, eplerenone, and amiloride.

#### 2.4.4. Carbonic Anhydrase Inhibitors (CAIs)

- Mechanisms of Action: Carbonic anhydrase inhibitors primarily target carbonic anhydrase isoenzyme II, which is found in the kidneys, eyes, and other tissues. The inhibition of carbonic anhydrase leads to several physiological effects:
  o Diuresis: In the kidneys, carbonic anhydrase inhibitors reduce bicarbonate reabsorption, leading to increased bicarbonate and water excretion, making them useful in conditions like edema and metabolic alkalosis.
  o Reduction of Intraocular Pressure: In the eyes, CAIs decrease the production of aqueous humor, making them a cornerstone in the treatment of glaucoma.

#### 2.4.5. Therapeutic Applications

- Edema: Systemic CAIs, like acetazolamide and methazolamide, are employed to manage edema in congestive heart failure, nephrotic syndrome, and high-altitude sickness.
- Metabolic Alkalosis: CAIs can be used to correct metabolic alkalosis by increasing renal bicarbonate excretion.

#### 2.4.6. Side Effects and Considerations

- Electrolyte Imbalances: CAIs can lead to hypokalemia and metabolic acidosis due to excessive bicarbonate excretion.
- Renal Stones: Prolonged use of CAIs may increase the risk of developing kidney stones, particularly in patients prone to stone formation.
- Sulfonamide Allergies: Some CAIs, such as acetazolamide, contain a sulfonamide moiety, which can lead to allergic reactions in individuals with sulfonamide allergies.

### 2.5. Anatomy of the Nephron and the Loop of Henle

To comprehend how loop diuretics work, it is essential to have a basic understanding of the anatomy of the nephron, the functional unit of the kidneys. The nephron consists of various segments, and one of the key segments is the loop of Henle. This U-shaped tubule is divided into two limbs: the descending limb and the ascending limb.

The ascending limb further differentiates into the thin and thick ascending limbs. The thick ascending limb is the primary site of action for loop diuretics.

### 2.6. Sodium-Potassium-Chloride Cotransporter (NKCC2)

The thick ascending limb is lined with specialized cells that express a key transporter known as the Sodium-Potassium-Chloride Cotransporter 2 (NKCC2). This cotransporter actively reabsorbs sodium, potassium, and chloride ions from the tubular fluid into the kidney cells. The movement of these ions across the cell membrane is essential for maintaining electrolyte balance in the body.

### 2.7. Mechanism of Loop Diuretics

Loop diuretics, including drugs like furosemide, bumetanide, and torsemide, exert their effects by specifically inhibiting the NKCC2 transporter in the thick ascending limb of the loop of Henle.

1. Inhibition of NKCC2: Loop diuretics competitively inhibit the NKCC2 transporter. They do this by binding to the chloride-binding site of the cotransporter. As a result, the transporter’s ability to reabsorb sodium, potassium, and chloride ions is significantly impaired.
2. Reduced Sodium Reabsorption: By inhibiting NKCC2, loop diuretics disrupt the normal process of sodium, potassium, and chloride reabsorption. This reduction in sodium reabsorption leads to a decrease in the osmotic gradient within the nephron, thus preventing the passive reabsorption of water that normally follows sodium reabsorption.
3. Increased Urine Output: The disrupted reabsorption of sodium and other ions results in a higher concentration of these ions in the tubular fluid. This increased osmotic load in the nephron prevents the reabsorption of water, promoting diuresis.

### 2.8. Pharmacokinetics and Dose-Response

#### 2.8.1. Bioavailability

- Furosemide, when administered orally, exhibits limited and highly variable bioavailability [73]. When kidney function is preserved, intravenous furosemide doses are almost twice as potent on a per-milligram basis as oral doses. In acute decompensated heart failure, a higher peak level may be required, and an intravenous dose may be more effective.
- Torsemide’s bioavailability can reach or exceed > 90% in patients with renal insufficiency, liver cirrhosis, and heart failure [74]. Torsemide’s bioavailability remains unchanged with food intake compared to the other two loop diuretics [75]. Torsemide’s peak serum concentration is similar to the other two substances but has the longest half-life of approximately 3.5 h vs. 1 h for furosemide and 2 h for bumetanide [76]. Passive venous congestion in HF patients can lead to gut edema, which can cause great variability in the diuretic effect, mainly of furosemide [77] due to malabsorption.

#### 2.8.2. Onset and Duration of Action

- The onset of action is rapid, typically within 30 min of administration.
- The duration of action is relatively short, usually around 4 to 6 h, necessitating multiple daily dosing.

#### 2.8.3. Dose–Response Curve

- Loop diuretics exhibit a steep dose–response curve, especially at lower doses.
- Lower doses of loop diuretics can cause a significant increase in diuresis, leading to pronounced sodium and water excretion.
- As the dose increases, the diuretic effect reaches a plateau, and further increases in dose may not significantly enhance diuresis but may increase the risk of adverse effects.

## 3. Diuretics in Heart Failure: Historical Perspective

The first ever HF case described belonged to 3500-year-old mummified remains found in the Valley of the Queens by the Italian Egyptologist Ernesto Schiaparelli [78]. Andreas Nerlich, a pathologist from Germany who performed the histologic examinations of the lungs, concluded, by exclusion, that the leading cause of death was pulmonary edema, likely due to HF [79].

Over the centuries, many civilizations have managed to describe the presence of fluid accumulation but without any understanding of the cause behind it [80], not making the connection between the symptom and the heart. The breakthrough occurred in 1918 when E.H. Starling [81] published his ‘Law of the Heart’. The demonstration that increasing end-diastolic volume enhances cardiac performance contradicted the 19th-century view that dilatation weakened the heart. Until the 1980s, the treatment was based on fluid restriction, rest, and the use of digitalis and diuretics, underlying the clear orientation of the scientific community towards kidney function rather than that of the heart. HF was finally recognized as a neuroendocrine disease in the 1980s and treatment with diuretics, vasodilators, and inotropes was put under discussion as it would keep the patient hostage in the vicious circle of the endocrine response present in HF [82].

The goal of keeping the patient in a euvolemic status remains, and this is exactly the treatment given when the patient decompensates, besides the optimal medical treatment that has been discovered throughout the years with potent agents like ACE inhibitors, ARNIs, b-blockers, MRAs, and SGLT 2 inhibitors. Although routine diuretic treatment of HF may appear uncomplicated, questions have arisen about the optimal use of diuretics, particularly in settings of ADHF and diuretic resistance (DR).

### 3.1. Challenges in Diuretic Therapy

Heart–kidney disorders caused by variable etiologies and precipitated by factors such as hemodynamic, neurohormonal, and inflammatory disorders can lead to cardiorenal syndrome (CRS) [83]. The clinical profile is characterized by decreased glomerular filtration, sodium avidity, and diuretic resistance (DR) [84].

### 3.2. Diuretic Resistance (DR)

Mortality, pump failure death, and sudden death present independent associations with diuretic resistance (DR). It may be defined as a non-satisfactory rate of diuresis/natriuresis despite an adequate diuretic regimen [85]. The diuretic resistance definition includes persistent congestion, despite adequate and escalating doses of diuretic agents equivalent to ≥80 mg/day furosemide; the amount of sodium excretion as a percentage of filtered load below 0.2% and failure to excrete at least 90 mmol of sodium within 72 h of a 160-mg twice-daily dose of furosemide. Other proposed parameters include weight loss achieved per 40 mg of furosemide or equivalent; net fluid loss per milligram of loop diuretic agent; and natriuretic response to furosemide as the urinary sodium-to-urinary furosemide ratio [86]. In HF patients, the prevalence of diuretic resistance (DR) is estimated at 20–30% [87]. It is vital to differentiate the homeostatic mechanism of the kidneys to protect themselves from a hypovolemic status and present a poor response to diuretics even in patients naive to diuretics [88]. Diuretic efficiency integrates the diuretic response in the context of the loop diuretic dose, dividing fluid output, weight change, or sodium output by the loop diuretic dose administered [89]. Diuretic efficiency is underscored in clinical practice since a modest response to a low-dose diuretic can result in good diuretic efficiency that is clinically unimportant if inadequate to bring the patient into a euvolemic status. It was proposed to be a mechanism of resistance according to anatomic location and significance [76]. When extra tubular, the mechanism can be venous congestion, increased intra-abdominal pressure or kidney vasoconstriction and hypoperfusion, decreased cardiac output, hypoalbuminemia, and high sodium intake. Even though gut edema and low duodenal blood flow do not typically affect furosemides’ oral bioavailability, they slow absorption, leading to reduced peak plasma levels, and therefore contribute to diuretic resistance. When tubular, it can be divided into the loop of Henle or the post-loop of Henle. In the former, an inadequate loop diuretic dose or rightward shift in the loop diuretic dose response curve should be checked, while for the latter compensatory distal tubular sodium reabsorption, hypochloremic alkalosis or specific transporters should be controlled [85]. Finally, the extent of natriuresis following a defined dose of diuretics decreases over time, even in normal subjects. This is called the ‘braking phenomenon’, and it is the result of both hemodynamic changes in the glomerulus and adaptive changes in the distal nephron. Loop diuretics are ‘threshold drugs’. The dose–response curve is shifted downwards and right due to heart failure. In other words, a higher dose of loop diuretics is needed in order to achieve the same level of sodium excretion.

The clinical presentation of diuretic resistance consists of insignificant relief of symptoms, further decompensation of heart failure besides the in-hospital treatment, increased mortality post-discharge, and up to three times higher rate of rehospitalization [86]. In the Acute Decompensated Heart Failure National Registry (ADHERE), 33% of the 50,000 patients enrolled that were treated with conventional diuretics lost around 2.3 kg, 16% gained weight while in hospital, and half of them were discharged with persistent congestion [90]. Moreover, in the Diuretic Optimization Strategies Evolution (DOSE) trial, 42% of participants with acute heart failure reached the end point of death or an unprogrammed visit to the hospital at 60 days irrespective of the treatment followed [91].

### 3.3. Treatment Strategies to Tackle DR

Once initiated, the effect of diuretic treatment needs to be monitored. For this purpose, an indicator needs to be used easily in daily clinical practice. There are two indicators that are used currently, the net fluid output and body weight changes. Weight assessment is technically challenging, and fluctuations seen in weight during hospitalization might not represent changes in volume redistribution. Further more, there is no clear correlation between fluid output and weight loss [92].

#### 3.3.1. Loop Diuretics

Intravenous loop diuretics exert their effect within the first couple of hours, and a return to baseline sodium excretion is noticed by 6–8 h. In this timeframe, early evaluation of the diuretic response can take place and will identify patients with a poor diuretic response [89,93]. It is known that thiazide and thiazide-like diuretics may partially overcome distal increased sodium avidity accompanied by chronic loop diuretic use [94]. In contrast to conventional knowledge, more recent evidence does support the effectiveness of thiazides in patients with a reduced glomerular filtration rate (<30 mL/min) [95].

In the DOSE-AHF trial, high loop diuretic dose, defined as 2.5 times the home dose and not less than 80 mg of furosemide per day, had a more favorable effect than the equal-to-home dose and this led to clinical improvement with dyspnea relief and a decrease in body weight and extravascular volume [71]. Renal dysfunction, defined as an increase in creatinine by more than 0.3 mg/dL, occurred more in the high-dose group. However, this increase did not affect the outcome as was shown by a post-hoc analysis of the DOSE-AHF trial [96]. Furthermore, a better outcome was seen in the high-dose group when adjusted for the total amount of loop diuretics received, suggesting that the adequacy of loop diuretic dosing to reach the ‘ceiling’ threshold is key [97]. The individual ceiling dose in each patient is difficult to determine and can be influenced by many factors, such as non-naivety with loop diuretics, body composition, the extent of volume overload, and renal function. Nonetheless, intravenous doses ranging between 400 and 600 mg furosemide vs. 10–15 mg bumetanide are generally considered the maximal total daily dose. When exceeded, additional natriuresis should be expected but this will lead to an increase in the side effects. Intravenous loop diuretics should be administered as soon as possible since early loop diuretic administration is associated with lower in-hospital mortality [98]. In the DOSE-AHF trial, no difference was seen in the primary endpoint between continuous or bolus infusion. If bolus infusion is chosen, doses should be administered in at least 6 h intervals to maximize the time above the natriuretic threshold and to avoid rebounding sodium retention [99].

Over the years, many efforts have been made to decrease both the resistance and the side effects of LD. Another well-investigated approach is that of changing the diuretic agent to torasemide. It is known to have the longest half-life at 3 to 4 h and can be as long as 5 to 6 h in patients with renal/hepatic dysfunction or heart failure. Bumetanide and torsemide exhibit higher and more consistent oral bioavailability (>90%) and do not exhibit absorption-limited kinetics, making oral and intravenous doses more comparable. In a recent meta-analysis, Miles et al. described a reduction in intermediate-term heart failure readmissions and improvement in the New York Heart Association class driven by torsemide compared with furosemide, which was not associated with a reduced mortality risk [100]. The TRANSFORM HF trial recruited 2859 participants hospitalized with heart failure and directly compared the novel loop diuretic torsemide (*n* = 1431) with furosemide (*n* = 1428) with investigator-selected dosages. Among patients discharged after hospitalization for heart failure, torsemide compared with furosemide did not result in a significant difference in all-cause mortality over 12 months [101]. Similar results were seen also in the ASCEND-HF trial where furosemide was also compared with torsemide and showed that torsemide use was not associated with significantly improved outcomes. However, in this trial, patients receiving torsemide had more comorbidities than those receiving furosemide. The landmark study on torsemide is the TORIC study, which compared torsemide to furosemide and found that after an average of 9 months, there was a significant 51.5% reduction in the risk of overall mortality, a 59.7% reduction in cardiac mortality, and a significant improvement in functional status within the torsemide group [102]. Unfortunately, the limitations of the study design included that they did not proceed to randomization, the sample population was mainly rural non-hospital based, and the use of other standard HF-pharmacotherapies such as beta-blockers and ACE inhibitors was low (~9.5% and ~30%, respectively).

#### 3.3.2. Mineralocortiocoid

Mineralocorticoid antagonists such as spironolactone improve mortality in heart failure with a reduced ejection fraction but need to be used at low doses of 25 mg in order to avoid hyperkalemia. Several small studies suggested that when mineralocorticoid antagonists are given in higher doses, called “natriuretic doses”, they might improve decongestion in ADHF [103]. The ATHENA study randomized 360 patients with ADHF and congestion to 96 h of spironolactone (100 mg daily) or placebo, but with a low dose of spironolactone continued [104]. Spironolactone did not improve either the primary endpoint of decongestion, measured by the change in NT-proBNP, or secondary endpoints, including symptom amelioration and decongestion. In contrast to the anticipated increase in potassium levels, the plasma potassium concentration was not affected, suggesting incomplete mineralocorticoid receptor blockade.

#### 3.3.3. Carbonic Anhydrase Inhibitor

As described above, one of the targets in heart failure is sodium reabsorption in the proximal tubules. Firstly, in a state of decompensated heart failure, sodium is reabsorbed mostly in the proximal nephron. Secondly, greater delivery of chloride to the macula densa cells increases, leading to a decrease in renin production, which reduces neurohumoral activation. Third, endogenous natriuretic peptides will possibly regain their cardioprotective effects. The carbonic anhydrase inhibitor acetazolamide acts in the proximal tubules inhibiting sodium reabsorption. An observational study in patients with decompensated heart failure and significant fluid overload showed that adding acetazolamide (500 mg intravenous bolus on top of loop diuretic) improved the loop diuretic response with approximately 100 mmol Na^+^ excreted per 40 mg of furosemide dose equivalents [105]. This synergic effect of acetazolamide with loop diuretics was also observed in a small, randomized trial with 24 patients, presenting with acute fluid overload resistant to loop diuretic therapy [106]. A multicenter, randomized, double-blind, clinical trial of the diuretic effects of Acetazolamide in Decompensated heart failure with Volume Overload (ADVOR) investigated whether acetazolamide can improve the efficiency of loop diuretics leading to faster and more efficient decongestion in ADHF. A total of 519 patients underwent randomization. In total, 108 of 256 in the treatment arm (42.2%) were successfully decongested as compared with 79 out of 259 (30.5%) in the placebo group (risk ratio, 1.46; 95% confidence interval [CI], 1.17 to 1.82; *p* < 0.001). The death rate and the rehospitalization rate were similar in both groups (29.7% vs. 27.8%) The treatment group had higher urine output and natriuresis, presenting an overall better diuretic effect. Adverse events, expressed by worsening kidney function, hypokalemia, and hypotension, were similar in both groups [107].

#### 3.3.4. SGLT2 Inhibitors

Sodium-glucose cotransporter 2 (SGLT2) inhibitors are a novel glucose-lowering treatment that blocks the SGLT2 protein, which is located in the proximal convoluted tubule of the nephron in type 2 adult patients. The substances are canagliflozin, dapagliflozin, and empagliflozin [108]. The Empagliflozin Outcome Event Trial in Type 2 Diabetes Mellitus Patients-Removing Excess Glucose (EMPA-REG OUTCOME) among patients with cardiovascular disease history indicated a significant reduction in the composite risk of cardiovascular death, myocardial infarction, or stroke by 14%. Overall, the risk of all-cause mortality was reduced by 32% during a follow-up period of 3.1 years [109]. Whether SGLT2 inhibitors provide clinical benefits in patients with AHF is being thoroughly explored. A total of 1831 patients took part in three different trials with their baseline characteristics mostly similar between interventional and control groups. The drug of choice was Empagliflozin in EMPULSE [110] and EMPA-RESPONSE-WHF [111] and Sotagiflozin in SOLOIST-WHF [112]. Compared with the placebo group, the risk of mortality was reduced by 27% in the intervention group (RR: 0.73, 95% CI: 0.49–1.09, *p* = 0.12, I2 = 18%). The mortality risk reduction was 15% in patients with Acute Decompensated Congestive Heart Failure (ADCHF) who took SGLT2 inhibitors compared to placebo (RR: 0.85, 95% CI: 0.62–1.15, *p* = 0.39, I2 = 0%). Compared to the placebo group, the intervention group had a significant risk reduction in Heart Failure Events (HFEs) of 62% (RR: 0.66, 95% CI: 0.58–0.75, *p* < 0.0001, I2 = 0%), defined as a hospitalization or visits to the emergency department, or an outpatient visit necessitating the intensification of treatment. Serious events were slightly lower in the intervention group by 15%, demonstrating a favorable safety profile in the three SGLT2 trials in acute heart failure (RR: 0.85, 95% CI: 0.70–1.03, *p* = 0.1, I2 = 44%). By the end of 2022, 15 clinical trials will have been conducted, testing the efficacy and safety of SGLT2 inhibitors on heart failure, diabetes mellitus type 2, acute myocardial infarction, and chronic kidney disease. The controlled substances are Empagliflozin of 10 and 20 mg, Dapagliflozin of 10 mg, and Canagliflozin. Control and group standard care consist of either placebo or loop diuretics, vasodilators, inotropic agents, digoxin, and/or vasopressors.

#### 3.3.5. Miscellaneous Approaches (Oral Vasopressin-2 Receptor Antagonist, Hypertonic Solutions, Dopamine)

Hyponatremia, reflecting water accumulation, is common in heart failure patients and is a poor prognostic indicator [113]. The oral vasopressin-2 receptor antagonist tolvaptan inhibits the action of antidiuretic hormone and increases free water excretion [114]. The EVEREST study, which evaluated hospitalized heart failure patients (with or without hyponatremia), did not demonstrate the superiority of tolvaptan over placebo in terms of long-term clinical outcomes. However, a beneficial effect on volume status and symptoms was observed on the initial treatment days [115]. Smaller trials focused on tolvaptan use in patients with lower serum sodium levels to achieve short-term decongestion did not show significant improvement in symptoms or clinical outcomes, despite leading to greater weight and fluid loss [116].

A randomized, single-blind study evaluated the effects of the combination of high-dose furosemide and small-volume hypertonic saline solution (HSS) infusion in the treatment of refractory New York Heart Association (NYHA) class IV CHF and a normal sodium diet during follow-up [117]. Patients were randomized into two groups. Patients in group 1 received an intravenous (IV) infusion of furosemide (500–1000 mg) plus HSS twice a day for 30 min. Patients in group 2 received an IV bolus of furosemide (500–1000 mg) twice a day, without HSS, during a period lasting 6 to 12 days. The results showed an improvement in quality of life, a delay in upscaling diuretic treatment, and a trend toward decreasing mortality.

When renal blood flow decreases, it contributes to sodium retention in ADHF. The proposed mechanism is limited Na+ filtration, increased Na+ reabsorption, and reduced renal diuretic delivery to the proximal tubule. Dopamine increases renal blood flow and was shown to cause urinary Na+ excretion at low doses [118] and therefore enhances natriuresis. The ROSE-AHF study randomized 360 patients hospitalized for ADHF with impaired renal function to furosemide plus either dopamine infusion (2 μg/kg/min), nesiritide (0.005 μg/kg/min), or placebo [119]. Urine volume or changes in cystatin C levels for 72 h were not affected by the two drugs. Dopamine infusion was associated with tachycardia (7% for dopamine vs. 1% for placebo, *p* > 0.001), even at this low dose. A post hoc subgroup analysis suggested that the low-dose dopamine effect could be different according to the heart failure subtype; in patients with heart failure with a reduced ejection fraction (HFrEF), dopamine may improve decongestion and prognosis [120].

## 4. Ultrafiltration Strategy (UF)

For years, the concept of a rapid decongestant performed mechanically by an ultrafiltration (UF) device has been under thorough investigation. UF presents many advantages over the classic diuretic treatment. These consist of precise control of the rate and amount of fluid removal, restoration of fluid responsiveness, removal of isotonic plasma water, no effect on the plasma concentration of potassium and magnesium, and finally, it does not exert direct neurohormonal activation. The disadvantages of the method are the need for anticoagulation, a peripheral or central venous catheter, and an extracorporeal circuit [121]. UNLOAD, CARRESS-HF, CUORE, and AVOID-HF are trials that investigated the role of UF in Acutely Decompensated Congestive Heart Failure (ADCHF). The key lessons from these trials are that UF can restore diuretic agent responsiveness, but overly aggressive fluid removal can convert nonoliguric renal dysfunction into oliguric failure and dialysis dependence.

The UNLOAD (UF vs. IV Diuretics for Patients Hospitalized for Acute Decompensated Heart Failure, *n* = 200) trial [122] was a multicenter, single-session, UF therapy for ADHF within 24 h. The trial showed that, compared with patients receiving intravenous (iv) Loop Diuretics (LD), those randomized to the ultrafiltration arm had greater weight and net fluid loss at 48 h and a 53% reduction in the 90-day risk of hospitalization and unscheduled visits for heart failure (*p* = 0.0037). In decompensated HF, UF can more safely produce weight and fluid loss than IV diuretics, reduces 90-day resource utilization for HF, and is an effective alternative therapy.

In contrast to the results of the UNLOAD trial, which tested the effects of early decongestive strategies, the CARRESS-HF (Cardiorenal Rescue Study in Acute Decompensated Heart Failure, *n* = 188) trial [123] showed that a stepped pharmacologic therapy algorithm was both superior and safer than a fixed 200 mL/h UF rate for the preservation of renal function at 96 h. The use of diuretics was superior to a strategy of UF for the preservation of renal function at 96 h, with a similar amount of weight loss on the two approaches. UF was associated with a higher rate of adverse events.

The AVOID-HF (Aquapheresis vs. IV Diuretics and Hospitalization for HF, *n* = 227) trial [124] showed that the Adjustable Ultrafiltration (AUF) group, compared with the Adjustable Loop Diuretic (ALD) group, had a non-statistically significant trend toward a longer time to first HF event after index hospitalization, significantly fewer patients rehospitalized, and shorter hospitalization times for HF or CV causes at 30 days. Whereas 90-day mortality did not differ between groups, the number of patients experiencing an adverse event of special interest or a serious product-related side effect was greater in the AUF than in the ALD group. The study was prematurely terminated by the sponsor. Nevertheless, the results of the AVOID-HF trial suggest that decongestion with UF requires careful evaluation of the benefit of reducing HF rehospitalizations with the risk of UF-related adverse events.

The CUORE trial [125], a small (*n* = 56), prospective, randomized, unblinded study, compared ultrafiltration and standard medical treatment. It did not include patients with acutely decompensated heart failure or cardiogenic shock. Moreover, randomization took place 24 h post admission, and fluid removal could not exceed 75% of the estimated initial weight increase. The intravenous dosage of diuretics that started before randomization was left unchanged in both groups.

## 5. Guidelines

According to the ESC guidelines for heart failure, diuretics are recommended in patients with congestion and both HFrEF and HFmrEF, with a class I level C recommendation, in order to alleviate symptoms and signs. UF still searches for its place in HF patients, as it is recommended in patients with advanced HF when in refractory volume overload, which is unresponsive to diuretic treatment with a class IIb indication level C. Renal replacement therapy should be considered in patients with refractory volume overload and end-stage kidney failure with a class IIa recommendation level C [18].

## 6. Conclusions

Multiple factors can contribute to the accumulation and redistribution of body fluid to the interstitial and intravascular compartments, often leading to volume overload and organ congestion. The renal retention of sodium and water is an early response mechanism contributing to fluid accumulation. The skillful use of diuretic therapy remains fundamental to HF management. The optimal assessment of volume status in HF patients is vital, particularly during the early management of the disease. LD is frequently used as the initial therapy to treat HF patients with fluid overload. Unfortunately, diuretics can have limited effectiveness due to several factors, such as underlying acute kidney injury, that contribute to diuretic resistance. UF and renal replacement therapies are often required for optimal volume management in patients with fluid overload as a bail-out treatment. It is of paramount importance to successfully estimate patients’ fluid status and set clear treatment goals. These goals seem to be achieved faster and more efficiently by ultrafiltration.

## Figures and Tables

**Table 1:** HF types according to Left Ventricular Ejection Fraction (LVEF).

Types of Heart Failure	Criteria
Heart Failure with reduced ejection fraction (HFrEF)	Symptoms ± Signs
	LVEF ≤ 40%
Heart Failure with mildly reduced ejection fraction (HFmrEF)	Symptoms ± Signs
	LVEF 40–49%
Heart Failure with preserved ejection fraction (HFpEF)	Symptoms ± Signs
	LVEF ≥ 50%

**Table 2:** NYHA functional classification of HF.

Class I	No limitation of physical activity. Ordinary physical activity does not cause undue breathlessness, fatigue, or palpitations.
Class II	Slight limitation of physical activity. Comfortable at rest, but ordinary physical activity results in undue breathlessness, fatigue, or palpitations
Class II	Marked limitation of physical activity. Comfortable at rest, but less than ordinary activity results undue breathlessness, fatigue, or palpitations.
Class IV	Unable to carry on any physical activity without discomfort. Symptoms at rest can be present. If any physical activity is undertaken, discomfort is increased.

**Table 3:** Light’s criteria on distinguishing the pleural effusion into exudate or transudate.

Light’s criteria
An effusion with any of the following characteristics is classified as an exudate
pleural: serum ratio > 0.5 pleural: serum LDH ratio > 0.6 pleural LDH > 2/3 of the upper limit of normal for the serum
An effusion with none of these characteristics is classified as a transudate.

**Table 4:** Classification of diuretics according the site and mechanism of action and their effects on electrolytes.

Diuretics Classification	Drugs Name	Site of Action	Mechanism of Action	Effects on Electrolytes
Loop diuretics	Furosemide Torasemide Bumetanide	Thick ascending limp of the loop of Henle	Inhibition of NaCl and the Na-K-2Cl cotransporter	↓ K^+^, Na^+^ in blood ↑ Bicarbonate excretion in urine and cause metabolic acidosis
Thiazide diuretics Thiazide-like diuretic	Hydrochlorothiazide Chlorthalidone Amiloride Clorapamide Indapamide	Distal tubule Additional proximal tubular action	Inhibition of NaCl cotransport Vasodilator	↓ K^+^, Na^+^, Mg^2+^ ↑ Ca^+2^ and uric acid blood level ↓ Cl^−^
Carbonic anhydrase inhibitors	Acetazolamide Dorazolamide Methazolamide Dichlorphenamide	Proximal convoluted tubule	Inhibition of carbonic anhydrase	↓ K^+^, Na^+^ in blood ↑ NAHCO3^−^ excretion in urine and cause metabolic acidosis

**Table 5:** Extrarenal effects and important side effects of diuretics.

Diuretics	Extrarenal Effects	Common or Important Side Effects
*LD*	↑ Venous capacitance ↑ Systemic vascular resistance ↓ Cardiac preload if chronically used	Ototoxicity Lipid abnormalities Rashes Hyperuricaemia Hyperglycaemia Dehydration
*THZ*	↑ Venous capacitance May be dose related	↑ LDL and triglycerides (may be transient) Hyperuricaemia Impotence Pancreatitis Rashes
*MRA*	*Antiandrogenic*	*Hyperkalaemia*
*CAI*	Raised level CO_2_ in brain and lowering of pH, leading to seizure threshold. Lowering intraocular tension Decreased gastric HCl and pancreatic NAHCO3^−^ secretion	*Neuropathy*

## Data Availability

Not applicable.
